# Effect of steaming on the quality characteristics of jujube fruit: A study of physicochemical properties, antioxidant activity and processing suitability across multiple cultivars

**DOI:** 10.1016/j.fochx.2025.103108

**Published:** 2025-10-01

**Authors:** Hanbing Zhu, Jia Tian, Junguang Ning, Ruirui Dao, Fuxu Pan, Mingrui Chen, Zhuanzhuan Liu, Mingzhu Lu, Mengjun Liu, Changwei Ao, Zhihui Zhao

**Affiliations:** a College of Food Science and Technology, Hebei Agricultural University, Baoding, Hebei 071001, China; b College of Horticulture, Hebei Agricultural University, Baoding, Hebei 071001, China; c Research Center of Chinese Jujube, Hebei Agricultural University, Baoding, Hebei 071001, China

**Keywords:** Jujube (*Ziziphus jujuba* mill.), Steaming, Texture profile analysis (TPA), Antioxidant stability, Sensory evaluation

## Abstract

Selecting optimal cultivars is essential for steaming, an emerging consumption method. This study evaluated 52 cultivars to examine the effects of steaming on physicochemical and sensory properties. Multivariate analysis identified WD, X13YZ, and JXMZ as the most suitable for industrial processing. Steaming significantly altered color parameters and texture, with hardness decreasing by 91.86 %. Heat-sensitive nutrients declined markedly, including vitamin C (−29.18%), flavonoids (−30.04 %), and total phenols (−35.86 %). Sugar composition shifted, with substantial increases in fructose (+47.95 %) and glucose (+70.96 %), while sucrose changes were cultivar-specific (e.g., +244.37 % in NJDMZ). Antioxidant activity decreased overall, though cultivars such as FMG and MLC retained over 75 % hydroxyl radical scavenging capacity. Hierarchical clustering grouped cultivars into four classes, enabling targeted applications. These findings provide a basis for cultivar selection to balance nutritional value and sensory quality in steamed products.

## Highlights

• Steaming alters jujube color (L*, a*, b*) and texture (hardness, chewiness, adhesiveness).
• Heat-sensitive nutrients (vitamin C, phenols) decline, while sugars (fructose, glucose) increase.
• WD, X13YZ, JXMZ show superior processing quality and antioxidant retention.
• Hierarchical clustering classifies 52 cultivars into four groups for targeted industrial applications.

## Graphical abstract

**Figure:**
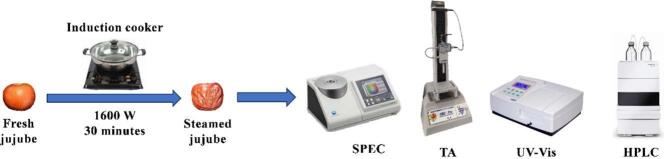

## 1 Introduction

Jujube (*Ziziphus jujuba* Mill.), a cornerstone of China's rural economy that accounts for more than 90 % of global production, is renowned for its abundance of bioactive compounds such as vitamin C and flavonoids, which contribute to antioxidant and immune-modulating effects (Cai et al., 2024; Jiang et al., 2025). Traditional processing methods, including drying and candying, dominate the industry. In contrast, steaming, a household technique that has recently gained popularity in northern China, remains largely unexplored at the industrial scale (Guo et al., 2015).

Steaming promotes sugar release in jujube fruit by hydrolyzing sucrose into fructose and glucose (Guo et al., 2015); however, it also accelerates the degradation of heat-sensitive nutrients, particularly vitamin C (Dorothy, Suinyuy, Lubaale, & Peter, 2023). Existing studies have generally focused on single cultivars or isolated parameters such as texture or nutrient content, without a systematic, multi-dimensional evaluation (Kong et al., 2022). This knowledge gap limits targeted cultivar selection for industrial-scale steaming.

In this study, we systematically evaluated 52 cultivars from China's National Jujube Germplasm Repository, representing the core genetic diversity of the species. Physicochemical, sensory, and antioxidant metrics were employed to (i) quantify steaming-induced changes in color, texture, and nutrients, (ii) identify cultivar-specific processing suitability, and (iii) establish a cultivar–quality prediction framework by integrating affiliation function analysis with hierarchical clustering. These results aim to provide a foundation for targeted industrial processing of jujube, enabling the selection of optimal cultivars for high-quality steamed products.

## 2 Materials and methods

### 2.1 Chemicals and reagents

Methanol (HPLC grade) was purchased from Sinopharm Chemical Reagent Co., Ltd. (Shanghai, China). Acetonitrile (HPLC grade) was purchased from Beijing Mairenda Technology Co., Ltd. (Beijing, China). Sodium hydroxide (NaOH, ≥98 %), sodium carbonate (Na_2_CO_3_, ≥99.5 %), sodium nitrite (NaNO_2_, ≥99 %), and aluminum nitrate nonahydrate (Al(NO_3_)_3_·9H_2_O, ≥98 %) were purchased from Tianjin Damao Chemical Reagent Factory (Tianjin, China). All other chemicals were of analytical grade and purchased from Sinopharm Group Co., Ltd. (Shanghai, China).

### 2.2 Plant material

Fifty-two cultivars of jujube fruit were harvested from the National Jujube Germplasm Repository in Cangxian County, Hebei Province, China, and the Fuping Experimental Station of Hebei Agricultural University (Table S1). The identification of all the cultivars was confirmed by Mengjun Liu (Professor of Hebei Agricultural University).

The 52 jujube cultivars selected for this study encompass major representative cultivars of the top six jujube-producing provinces in China. XG (Xingguang), a cultivar derived from ‘Junzao’ with resistance to jujube witches' broom disease, along with HZ (Huizao), serves as a key representative from Xinjiang. PZ (Pozao), ZHDZ (Zanhuangdazao), and JSXZ (Jinsixiaozao) are prominent cultivars in Hebei Province. MZ (Muzao) is widely cultivated and of considerable economic importance in both Shanxi and Shaanxi Provinces. Additionally, JSXZ (Jinsixiaozao) and YLXZ (Yuanlingxiaozao) are characteristic cultivars originating from Shandong Province. Furthermore, several cultivars with potential for processing applications have been selected, including FS (Fushuai), WHF (Wuhefeng), ZB (Zanbao), YG (Yueguang), FMG (Fengmiguan), LBY (Lengbaiyu), and WD (Wudeng). The inclusion of these cultivars ensures a comprehensive representation of the core jujube germplasm.

### 2.3 Steaming treatment and sample preparation

One hundred medium-sized jujube fruits per cultivar were randomly divided into two groups. One group served as the fresh control, while the other was steamed for 30 min at 1600 W using a C22-WH2202 induction cooker (Midea Group Co., Ltd., Foshan, China). Half of the intact fresh and steamed jujube fruits were used for color, texture, and sensory evaluations. The remaining samples were immediately frozen in liquid nitrogen, ground into a fine powder with a pre-chilled mortar and pestle, and stored at −80 °C until further analysis.

### 2.4 Determination of color characteristics of jujube fruit

The color characteristics were measured using a CM-5 spectrophotometer (Konica Minolta, Tokyo, Japan), and each group of samples was measured three times (Jiang et al., 2023).

### 2.5 Texture profile analysis (TPA)

Texture profile analysis (TPA) was performed using a texture analyzer (DE-100 g, Zhejiang Hongjingtian, China) equipped with a cylindrical probe. The test conditions were: initial force 100 g, test speed 30 mm/s, 30 % compression deformation, 60 mm lift height, and 1.5 s pause time. Hardness, springiness, adhesiveness, cohesiveness, gumminess, and chewiness were derived from the TPA curves (Contador, Shinya, & Infante, 2015).

### 2.6 Sensory evaluation analysis

Sensory evaluation was carried out by a trained panel of 10 members (5 males and 5 females, aged 18–50 years) selected from students and faculty members of Hebei Agricultural University (HAU). Prior to the evaluation, panelists underwent a training session to familiarize themselves with the sensory characteristics of jujube fruit and assess their sensory sensitivity. During the evaluation, pure water at room temperature was used as a neutralizer between samples. After steaming, samples were randomly presented, and evaluations followed a sequential protocol: visual inspection, olfactory assessment, and gustatory analysis (Lin et al., 2025; Yao et al., 2024). Sensory attributes were scored on a 10-point scale (Table S2), and they were fully informed about the purpose of this study and provided written consent to participate in the experiment. The study protocol was approved by the Ethics Review Committee of the College of Food Science and Technology, HAU, complying with human research ethics.

### 2.7 Determination of Vitamin C, Titratable Acid, Total Phenols, Flavonoids, Soluble Proteins, and Total Triterpenoids

Vitamin C (VC) content was determined following a modified protocol described by LO'AY et al. (Lo'ay & Dawood, 2019). Briefly, 0.5 g of jujube powder was mixed with 40 mL of 2 % (*w*/*v*) oxalic acid solution under low-temperature and light-protected conditions. The mixture was vortexed for 1 min, centrifuged at 12,000 ×*g* (Sorvall ST 8R, Thermo Fisher Scientific (China) Co., Ltd., Shanghai, China) for 5 min at 4 °C, and 10 mL of the supernatant was titrated with 2,6-dichlorophenol indophenol solution until a light pink endpoint persisted for 0.5 min. The vitamin C content was measured and expressed as g per kg. Each sample was analyzed in triplicate.

Titratable acidity was determined according to a modified method described by ZUO et al. (Zuo et al., 2024). Briefly, jujube powder (0.5 g) was extracted in 10 mL distilled water at 80 °C for 30 min with shaking. After cooling, adding 40 mL water and centrifugation (10,000 ×*g*, 5 min, 25 °C), 20 mL aliquot of the supernatant mixed with 2 drops of 1 % (*w*/*v*) phenolphthalein indicator, and titrated with 0.01 mol L^−1^ NaOH until a faint pink endpoint was reached. Results were expressed as percentage acidity, with blank correction and triplicate measurements.

Flavonoid content was determined according to a modified method described by LIU et al. (Liu, Lei, Guo, Yao, & Zeng, 2023). Briefly, 0.2 g of jujube powder was extracted with 10 mL of anhydrous ethanol using 60 W ultrasonic treatment for 30 min at 25 °C. A 1 mL aliquot of the supernatant was mixed with 0.6 mL of 5 % (*w*/*v*) NaNO_2_ solution, incubated for 6 min, followed by the addition of 0.4 mL of 10 % (w/v) Al (NO_3_)_3_ solution and another 6 min incubation. Finally, 2 mL of 4 % (w/v) NaOH solution was added, and the mixture was incubated for 30 min before measuring the absorbance at 511 nm. Each sample was analyzed in triplicate. The flavonoid content was expressed as grams of rutin equivalents per kilogram (g RE kg^−1^).

Total phenolic content (TPC) was quantified using a modified Folin-Ciocalteu method (Liu et al., 2023). Briefly, 0.2 g samples were ultrasonically extracted (60 W (KQ-500DE, Kun Shan Ultrasonic Instruments Co., Ltd., Jiangsu, China), 30 min, 25 °C) with 10 mL ethanol. The supernatant (0.15 mL) was reacted with 2.85 mL water, 0.5 mL Folin-Ciocalteu reagent, and 1.5 mL 7.5 % (*w*/*v*) Na₂CO₃ solution. After 120 min dark incubation, absorbance at 765 nm (TU-1810, Beijing Purkinje GENERAL Instrument Co., Ltd., Beijing, China) was measured. TPC was calculated using a gallic acid standard curve and expressed as g GAE kg^−1^. Measurements were performed in triplicate.

Soluble protein content was determined following a modified protocol described by LV et al. (Lv et al., 2022). Briefly, 0.5 g of jujube powder was combined with 50 mL of distilled water. The mixture was subjected to ultrasonication at 25 °C for 30 min at a power of 60 W. Subsequently, it was centrifuged at 12,000 ×*g* for 5 min. An aliquot of 0.1 mL of the supernatant was transferred into 5 mL of Coomassie Brilliant Blue G-250 reagent. The mixture was thoroughly mixed and allowed to stand for 2 min. Absorbance values were then measured at 595 nm using a UV spectrophotometer. Each sample was measured in triplicate in parallel, and the soluble protein content was expressed as grams per kilogram (g kg^−1^).

Total triterpene content was determined following a modified protocol described by XUE et al. (Xue, Wang, & Zhou, 2022). Briefly, 0.5 g of jujube powder was mixed with 10 mL of 95 % (*v*/v) ethanol by vortexing followed by sonication (60 W, 30 min, 25 °C) and then stored overnight at 4 °C. After centrifugation, 0.1 mL of the supernatant was evaporated in a 90 °C water bath. The residue was reacted with 0.8 mL of perchloric acid and 0.2 mL of 5 % (*w*/*v*) vanillin-glacial acetic acid solution in a 60 °C water bath for 20 min, then rapidly cooled in an ice-water bath for 3–5 min. Subsequently, 5 mL of glacial acetic acid was added, and the mixture was incubated for 10 min before measuring the absorbance at 548 nm. The total triterpene content was expressed as grams per kilogram (g kg^−1^), with all analyses performed in triplicate.

### 2.8 Determination of fructose, sucrose, and glucose content

Sample preparation was performed according to the method described by PU et al. (Pu et al., 2017). Briefly, jujube powder sample (5.0 g) was extracted with 100 mL distilled water at ambient temperature for 30 min using sonication, and the extract was centrifuged at 8000 ×*g* for 15 min. The supernatant was filtered with a 0.22 μm syringe filter for detection.

HPLC analysis was performed using an Agilent 1260 Infinity system (Agilent Technologies, Santa Clara, CA, USA) equipped with an NH_2_P-50 4E column (250 mm × 4.6 mm, 5 μm) and an evaporative light scattering detector (ELSD). The mobile phase consisted of acetonitrile and water (75:25, *v*/v) at a flow rate of 0.8 mL min^−1^. The injection volume was 10 μL, and the column temperature was maintained at 30 °C. The ELSD parameters were set as follows: evaporation temperature of 30 °C, nebulization temperature of 30 °C, and gas flow rate of 1.60 mL min^−1^. The total run time was 25 min.

### 2.9 Determination of antioxidant activity

Briefly, 0.5 g of jujube powder was extracted with 20 mL of 70 % (*v*/v) ethanol using ultrasonic treatment at 60 °C for 50 min. The mixture was centrifuged at 10,000 ×*g* for 5 min at room temperature, and the supernatant was collected as the sample extract.

ABTS free radical scavenging activity was determined according to a modified method described by LIN et al. (Lin et al., 2020). The ABTS free radical stock solution was generated by reacting K₂S₂O₈ (140 mmol L^−1^, 440 mL) with ABTS (7 mmol L^−1^, 25 mL) and incubating in the dark for 12–16 h, then diluted with ethanol to an absorbance of 0.700 ± 0.002 at 734 nm. Sample extract (0.2 mL) was mixed with 4.8 mL of this working solution and incubated in the dark at 25 °C for 15 min. The absorbance of the resulting mixture was measured at 734 nm and denoted as A₁. A blank control was prepared identically by replacing the sample extract with 0.2 mL of ethanol, and its absorbance was measured and denoted as A₀.

$$\;\text{ABTS free radical scavenging rate}=\frac{A0-A1}{A0}\times 100\%.$$

Hydroxyl radical scavenging activity was determined using the salicylic acid method (Sforcin, 2016). Briefly, 0.2 mL of the sample extract was reacted with 1 mL of 6 mmol L^−1^ FeSO₄ and 1 mL of 6 mmol L^−1^H₂O₂ (vortexed for 1 min; incubated for 10 min at 25 °C). Then, 1 mL of salicylic acid solution was added, and after vortexing and incubation for 60 min, the absorbance was measured at 510 nm (denoted as A₁). A blank control (A₀) used distilled water instead of the sample extract, and a negative control (A₂) replaced H₂O₂ with distilled water.

$$\;\text{Hydroxyl radical scavenging rate}=\frac{A0-A1+A2}{A0}\times 100\%$$

### 2.10 Comprehensive evaluation

Comprehensive evaluation was performed using the affiliation function method in fuzzy mathematics as described by SU et al. (Su et al., 2024). For each material, the affiliation value of each index was calculated, and the cumulative affiliation values were used to compare and rank the cultivars. The affiliation function value (AFV) was calculated as follows:

$$\left(\mathrm{AFV}=\right)\;\frac{X-\text{Xmin}}{\;\text{Xmax}-\text{Xmin}}\times 100\%$$

where X is the measured value of a specific index for a material, Xmax is the maximum value of that index across all materials, and Xmin is the minimum value of that index across all materials. For each cultivar, the affiliation values of all indicators were summed, and the average affiliation value was calculated. Cultivars with higher average affiliation values were considered superior.

### 2.11 Statistical analysis and visualization

Statistical analysis was performed using SPSS 22.0 (SPSS-IBM, Chicago, IL, USA). One-way analysis of variance (ANOVA) and post-hoc Tukey's honestly significant difference (HSD) test was conducted, and the significance level was set at *P* < 0.05. Heatmap visualization, correlation analysis, and hierarchical clustering dendrograms were generated using Origin 2022 (OriginLab Corporation, Northampton, MA, USA). All analyses were performed in triplicate, and calibration data were standardized to ensure accuracy.

## 3 Results and discussion

### 3.1 Phenotypic investigation

**Fig. 1:**
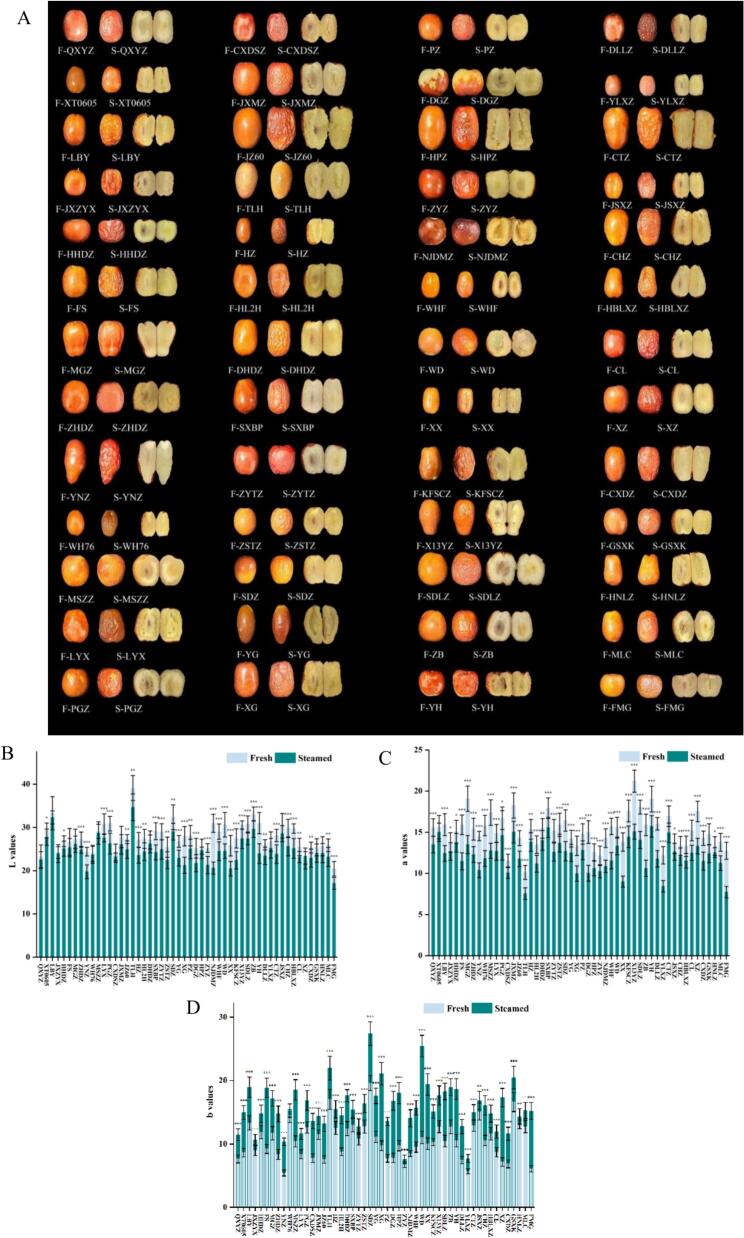
Phenotypic parameter changes before and after steaming: (A) Morphology, (B) L*, (C) a*, (D) b*. Significance levels: *P < 0.05, **P < 0.01, ***P < 0.001 (*t*-test).

To comprehensively assess phenotypic changes in jujube fruits before and after steaming, visual appearance was examined (Fig. 1A). Steaming induced pronounced wrinkling of the fruit peel. Longitudinal sections showed that 15 cultivars (QXYZ, JXZYX, MGZ, YNZ, MSSZ, PGZ, JXMZ, SXBP, ZYTZ, WHF, WD, SDLZ, ZB, DLLZ, and FMG) retained white flesh, while the remaining 37 cultivars displayed varying degrees of yellow coloration.

Steaming significantly altered epidermal color parameters. The L values decreased from 27.73 ± 1.98 (fresh) to 24.70 ± 1.90 (steamed), representing a 10.93 % reduction, indicating decreased brightness (Fig. 1B). The *a* values also declined significantly, from 15.19 ± 1.18 to 12.27 ± 1.03, corresponding to a 19.22 % reduction and reflecting diminished red pigmentation (Fig. 1C). This decline may result from degradation of heat-sensitive pigments (e.g., anthocyanins) and accumulation of phenolic oxidation products (Boateng & Clark, 2024), consistent with the observed post-steaming epidermal browning. In contrast, the *b* values generally increased, rising from 10.53 ± 0.85 to 15.85 ± 1.29, a 50.52 % increase (Fig. 1D). Notably, the 15 cultivars with white flesh (e.g., QXYZ, JXZYX) showed only minor increases in *b* values, whereas the 37 yellow-fleshed cultivars exhibited more pronounced increases.

This pronounced browning and darkening (decreases in L and a values) are likely driven by two main mechanisms: thermal degradation of anthocyanins and other heat-sensitive pigments, and enzymatic or non-enzymatic oxidation of phenolic compounds, resulting in the formation of melanoidins and other brown polymers (Boateng et al., 2024; J. Zhang, Zhang, Zhang, Xue, & Zhang, 2025). The cultivar-dependent variation in b values increases underscores the genetic diversity of pigment composition and stability among jujube cultivars. From a consumer perspective, cultivars with vibrant coloration (e.g., the 37 yellow-fleshed cultivars such as XT0605) are preferable for products where visual appeal is critical, whereas other cultivars may be more suitable for products with lower color requirements, such as fruit purees or concentrated juices. Overall, steaming induced marked cultivar-dependent color changes, highlighting the influence of thermal processing on fruit pigmentation. Importantly, the observed differences in color stability appear to be genetically encoded, likely involving the phenylpropanoid pathway. This genetic basis provides a valuable foundation for both the targeted selection of cultivars for specific processed products and breeding programs aimed at improving color retention.

### 3.2 Texture profile analysis

Texture is a critical determinant of jujube fruit quality, strongly influencing consumer acceptance and sensory evaluation. TPA, which simulates mastication and swallowing in the human oral cavity, provides objective measurements of key parameters, including hardness, springiness, adhesiveness, cohesiveness, and chewiness (Kong et al., 2022).

**Fig. 2:**
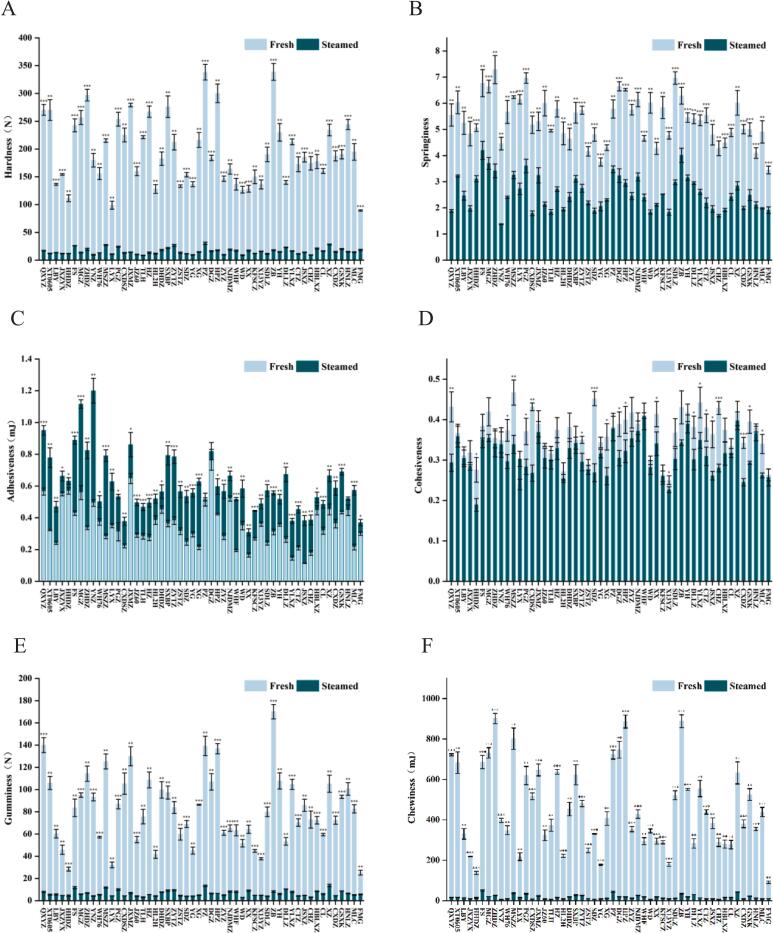
Texture profile analysis: (A) Hardness, (B) Springiness, (C) Adhesiveness, (D) Cohesiveness, (E) Gumminess, (F) Chewiness. Significance levels: *P < 0.05, **P < 0.01, ***P < 0.001 (*t*-test).

Hardness, reflecting the fruit's resistance to compression, is a primary indicator of chewing sensation and overall quality. Steaming led to a pronounced reduction in hardness (Fig. 2A). Fresh samples exhibited a mean hardness of 195.59 ± 9.26 N, compared with 15.93 ± 0.79 N in steamed samples, representing a 91.86 % decrease. Among fresh cultivars, FMG had the lowest hardness (89.39 ± 1.25 N), while ZB showed the highest (338.86 ± 15.05 N). After steaming, TLH exhibited the lowest hardness (8.61 ± 0.40 N), whereas PZ retained the highest (30.36 ± 2.03 N). The observed reduction in hardness may be attributed to enhanced activity of cell wall-degrading enzymes, such as pectin methylesterase (PME) and polygalacturonase (PG), which promote pectin de-esterification and polygalacturonic acid hydrolysis. These processes weaken cell wall integrity, leading to fruit softening during steaming (Sun et al., 2022).

Springiness, defined as the ability of fruit to regain its original shape after deformation, is a key parameter reflecting resilience and texture quality. Steaming significantly reduced springiness (Fig. 2B), with mean values decreasing from 5.39 ± 0.30 (fresh) to 2.56 ± 0.10 (steamed), a 52.50 % reduction. Among fresh cultivars, FMG exhibited the lowest springiness (3.45 ± 0.15), whereas ZHDZ showed the highest (7.29 ± 0.54). After steaming, YNZ had the lowest springiness (1.37 ± 0.02), while FS retained the highest (4.20 ± 0.35). The loss of resilience may be attributed to protein denaturation under high-temperature steaming (Li et al., 2024), which explains why cultivars with firmer pulp (e.g., ZHDZ) maintained higher springiness, while those with softer pulp (e.g., FMG) showed greater reductions.

Adhesiveness, representing the stickiness of fruit surfaces, reflects their ability to adhere to other materials and is a critical factor influencing processability, storage, and sensory attributes such as mouthfeel and handling. Steaming significantly increased adhesiveness (Fig. 2C), with mean values rising from 0.35 ± 0.02 mJ (fresh) to 0.61 ± 0.03 mJ (steamed), a 74.29 % increase. Among fresh cultivars, JSXZ exhibited the lowest adhesiveness (0.11 ± 0.00 mJ), whereas DGZ showed the highest (0.77 ± 0.07 mJ). After steaming, XX had the lowest adhesiveness (0.31 ± 0.02 mJ), while YNZ reached the highest (1.20 ± 0.08 mJ).

Cohesiveness, defined as the relative resistance during the second compression after initial deformation, reflects the ability of jujube fruit to maintain structural integrity during mastication. Steaming significantly reduced cohesiveness (Fig. 2D), with mean values decreasing from 0.37 ± 0.03 (fresh) to 0.31 ± 0.02 (steamed), a 16.22 % reduction. Among fresh cultivars, LYX exhibited the lowest cohesiveness (0.24 ± 0.01), whereas MSZZ showed the highest (0.47 ± 0.03). After steaming, HHDZ retained the lowest cohesiveness (0.19 ± 0.02), while WHF exhibited the highest (0.41 ± 0.03). The reduction in cohesiveness may be attributed to fiber loosening caused by water loss during steaming (Y. Zhang et al., 2024). Notably, cultivars such as PZ and KFSCZ showed minimal changes, suggesting that their compact pulp structures are more resistant to thermal degradation.

Gumminess, which represents the resistance of fruit tissue to deformation during mastication and its adhesion to oral surfaces, directly influences sensory perception. Steaming markedly reduced gumminess (Fig. 2E), with mean values declining from 81.81 ± 4.57 N (fresh) to 6.47 ± 0.40 N (steamed), a 92.10 % reduction. Among fresh samples, FMG showed the lowest gumminess (25.31 ± 2.17 N), whereas ZB exhibited the highest (170.11 ± 6.46 N). After steaming, WD retained the lowest gumminess (2.90 ± 0.04 N), while XZ showed the highest (13.78 ± 1.05 N). Cultivars with inherently low gumminess (e.g., HHDZ, FMG) exhibited relatively minor changes, indicating greater stability during thermal processing.

Chewiness, defined as the energy required to masticate food, is a key indicator of texture quality and sensory perception. It reflects the mechanical behavior of fruit during mastication, directly influencing chewability and overall consumer experience. Steaming significantly reduced chewiness (Fig. 2F), with mean values decreasing from 455.45 ± 22.19 mJ (fresh) to 17.45 ± 0.98 mJ (steamed), representing a 96.17 % reduction. Among fresh samples, FMG exhibited the lowest chewiness (90.81 ± 5.47 mJ), while ZHDZ showed the highest (901.60 ± 25.50 mJ). After steaming, CHZ displayed the lowest chewiness (5.30 ± 0.44 mJ), whereas FS retained the highest (50.17 ± 3.88 mJ). Cultivar-dependent variation was evident, with ZHDZ (901.60 ± 25.50 mJ fresh vs. 24.81 ± 1.60 mJ steamed) and HPZ (886.98 ± 32.41 mJ fresh vs. 18.48 ± 1.50 mJ steamed) exhibiting the most pronounced reductions. These findings suggest that steaming-induced cell wall degradation and structural softening (Bao et al., 2024) substantially impact pulp chewiness.

The drastic softening observed after steaming likely results from combined thermal and enzymatic cell wall disassembly (Kong et al., 2022; Sun et al., 2022). While this enhances consumer appeal by producing a softer, stickier, and sweeter product, it also reduces structural stability. Notably, cultivars such as PZ and KFSCZ demonstrated exceptional resistance to steaming-induced textural loss, making them ideal candidates for processing applications where pulp integrity is critical, such as canning.

### 3.3 Sensory evaluation analysis

**Fig. 3:**
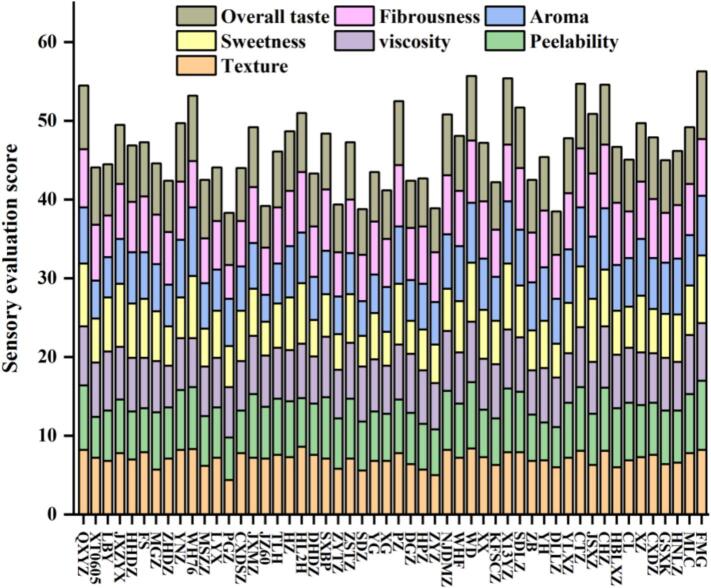
Sensory evaluation of jujube fruit from different cultivars.

Sensory analysis is essential for assessing consumer acceptability and the commercial potential of processed fruit products such as juices and functional beverages (Alizadeh Behbahani, Jooyandeh, Hojjati, & Ghodsi Sheikhjan, 2024; Echresh, Behbahani, Falah, Noshad, & Ibrahim, 2025; Kardooni, Alizadeh Behbahani, Jooyandeh, & Noshad, 2023; Mousanejadi, Barzegar, Alizadeh Behbahani, & Jooyandeh, 2023). Differences in jujube fruit quality among cultivars resulted in significant variation in sensory scores (Fig. 3). Texture varied notably: PGZ and ZYZ received the lowest scores, indicating a coarse texture likely to diminish flavor perception. Peelability also differed, with FMG achieving the highest score, whereas YH scored lowest, reflecting tightly adhered skin. Viscosity ranged from high in CHZ to low in ZHDZ. Sweetness strongly influenced overall flavor, with FMG being the sweetest cultivar, followed by X13YZ, while SDZ was the least sweet. Aroma intensity also varied, with WH76 exhibiting the strongest aroma and JZ60 the weakest. Fibrousness differed significantly: PGZ had the lowest score, while CHZ and JSXZ scored higher, suggesting a finer pulp texture. Overall taste scores ranked FMG highest, followed by X13YZ, whereas JZ60 and DLLZ scored lowest. Based on total sensory evaluation, FMG, WD, and X13YZ achieved the highest scores (all >55), while PGZ, DLLZ, SDZ, ZYZ, JZ60, and ZYTZ scored lowest (all <40). The remaining cultivars scored between 40 and 55. Sensory texture acceptance correlated closely with instrumental TPA. Cultivars preferred for their soft texture, such as FMG, exhibited significantly reduced hardness, springiness, and chewiness, while cultivars with coarser textures, such as PGZ, displayed higher hardness and springiness. These results confirm that TPA provides a reliable predictor of the sensory quality of steamed jujubes.

### 3.4 Nutritional composition

#### 3.4.1 Vitamin C (VC) content

**Fig. 4:**
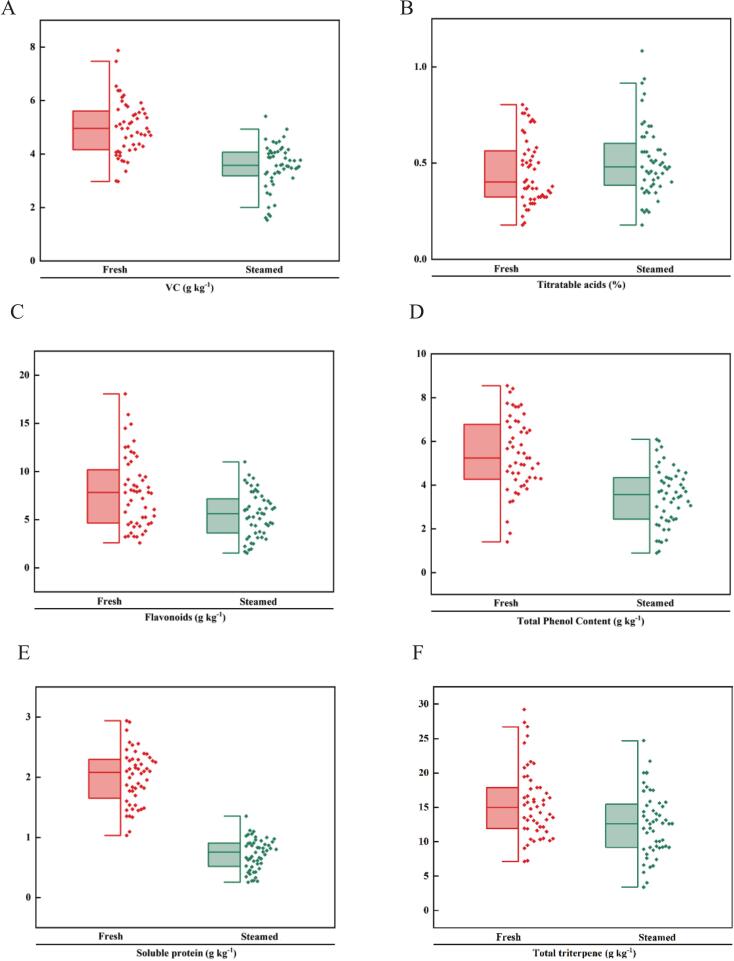
Changes in nutritional components of jujube fruit before and after steaming: (A) VC, (B) Titratable acids, (C) Flavonoids, (D) Total Phenol Content, (E) Soluble protein, (F) Total triterpene.

VC, an essential secondary metabolite in fruit development, possesses a dienol structure that confers strong antioxidant capacity but also makes it highly susceptible to degradation by heat, oxygen, and light through both enzymatic and non-enzymatic pathways. VC plays a critical role in human health by regulating redox reactions and supporting immune function. Fresh jujube samples contained significantly higher VC levels than steamed samples, with most cultivars exhibiting substantial losses during steaming (Fig. 4A, Table S3). The mean VC content declined from 4.97 ± 0.15 g kg^−1^ (fresh) to 3.52 ± 0.11 g kg^−1^ (steamed), corresponding to a 29.18 % reduction. This confirms that heat treatment is the primary postharvest factor contributing to VC degradation (S. K. Lee & Kader, 2000). This confirms that heat treatment is the primary postharvest factor contributing to VC degradation. Although most cultivars showed pronounced VC losses, a few, such as CXDZ and WD, demonstrated minimal reductions, suggesting superior retention capacity. Such cultivar-specific differences may be attributed to inherent variations in cellular composition and indicate the potential for selective breeding or cultivar choice to improve the nutritional quality of steamed jujube products. Among steamed samples, DGZ had the lowest VC content (1.53 ± 0.02 g kg^−1^), whereas HNLZ retained the highest (4.93 ± 0.12 g kg^−1^). Steaming conditions, including temperature, duration, and humidity, also significantly influenced VC stability, with higher temperatures and extended processing times accelerating degradation.

#### 3.4.2 Titratable acids

Titratable acids, composed of various organic acids, are major contributors to fruit acidity and play a central role in sensory quality (Wang, Jian, et al., 2025). Steaming significantly increased titratable acid content across all cultivar (Fig. 4B, Table S3). The mean value rose from 0.46 ± 0.03 % (fresh) to 0.51 ± 0.02 % (steamed), representing a 10.87 % increase. Among steamed samples, YNZ exhibited the lowest titratable acid content (0.18 ± 0.02 %), whereas FMG had the highest (1.08 ± 0.02 %). The increase in acidity may be attributed to the thermal degradation of complex compounds, such as polysaccharides and phenolics, into small-molecule organic acids. In addition, water loss and solute concentration during steaming likely contributed to the universal rise in titratable acids. This enhanced acidity not only intensifies tartness but also balances the increased sweetness, thereby adding complexity to the flavor profile of steamed jujube fruit (Fellows, 2017).

#### 3.4.3 Flavonoid and Total phenol content

Flavonoids and total phenols are key bioactive compounds with antioxidant, anti-inflammatory, and anticancer properties, contributing significantly to the health-promoting value of jujube. Steaming caused a marked reduction in flavonoid content across all cultivars (Fig. 4C, Table S3). Mean values declined from 7.89 ± 0.32 g kg^−1^ (fresh) to 5.52 ± 0.26 g kg^−1^ (steamed), a 30.04 % reduction. Among steamed samples, XX exhibited the lowest flavonoid content (1.53 ± 0.05 g kg^−1^), whereas SXBP retained the highest (11.00 ± 0.25 g kg^−1^). The exceptional stability of SXBP and certain other cultivars suggests the presence of phenolic compounds with greater thermal tolerance. The overall decline may be explained by solubilization of flavonoids and polyphenols, as well as the formation of phenolic–protein complexes during thermal processing (Bagchi et al., 2021). Similarly, total phenolic content decreased significantly after steaming (Fig. 4D, Table S3). Mean values dropped from 5.41 ± 0.07 g kg^−1^ (fresh) to 3.47 ± 0.08 g kg^−1^ (steamed), representing a 35.86 % reduction. Among steamed cultivars, JXZYX had the lowest phenolic content (0.89 ± 0.28 mg/g), while SXBP retained the highest (6.08 ± 0.10 g kg^−1^). The reduction is likely due to partial loss or structural transformation of water-soluble phenolic compounds under heat treatment (Bagchi et al., 2021).

#### 3.4.4 Soluble protein

Soluble proteins in jujube fruit contribute to nutrient supply, antioxidant activity, texture improvement, and stress resistance, while also participating in metabolic processes and the biosynthesis of functional compounds. Across all cultivars, soluble protein content showed a significant decline after steaming (Fig. 4E, Table S3). Mean values decreased from 2.00 ± 0.08 g kg^−1^ (fresh) to 0.72 ± 0.03 g kg^−1^ (steamed), representing a 64.00 % reduction. Among steamed cultivars, SDLZ exhibited the lowest soluble protein content (0.27 ± 0.02 g kg^−1^), whereas NJDMZ retained the highest (1.35 ± 0.02 g kg^−1^). This reduction is primarily attributed to heat-induced protein denaturation. Elevated temperatures cause unfolding of secondary and tertiary structures, exposing hydrophobic groups, which promotes intermolecular aggregation or precipitation and consequently reduces solubility (Zhou, Xu, Yu, & Wang, 2024).

#### 3.4.5 Total triterpene

Total triterpenes, known for their antioxidant, anti-inflammatory, immunomodulatory, and hypolipidemic properties, contribute substantially to the nutritional and functional value of jujube fruit. After steaming, triterpene content showed a bidirectional response across cultivars (Fig. 4F, Table S3). Seventeen cultivars exhibited significant increases in total triterpenes. For example, XG increased from 7.25 ± 0.52 g kg^−1^ to 15.87 ± 0.49 g kg^−1^, a 111.04 % rise. This enhancement may result from heat-induced disruption of cell wall and membrane structures, facilitating the release of bound triterpenoic acids (Liu, Lei, Guo, Yao, & Zeng, 2023). In contrast, 35 cultivars showed marked decreases. For instance, HZ declined from 25.38 ± 1.52 g kg^−1^ to 6.51 ± 0.35 g kg^−1^, representing a 73.35 % reduction. This reduction is likely linked to the oxidative decomposition of heat-sensitive triterpenes, particularly volatile sesquiterpenes (Li, Ullah, et al., 2023).

The bidirectional trend highlights a cultivar-specific response, reflecting the balance between the release of bound triterpenes and their thermal degradation (H. Y. Lee et al., 2024). Cultivars exhibiting net increases may serve as promising sources for triterpenoid extraction in industrial applications.

### 3.5 Fructose, glucose and sucrose content

**Fig. 5:**
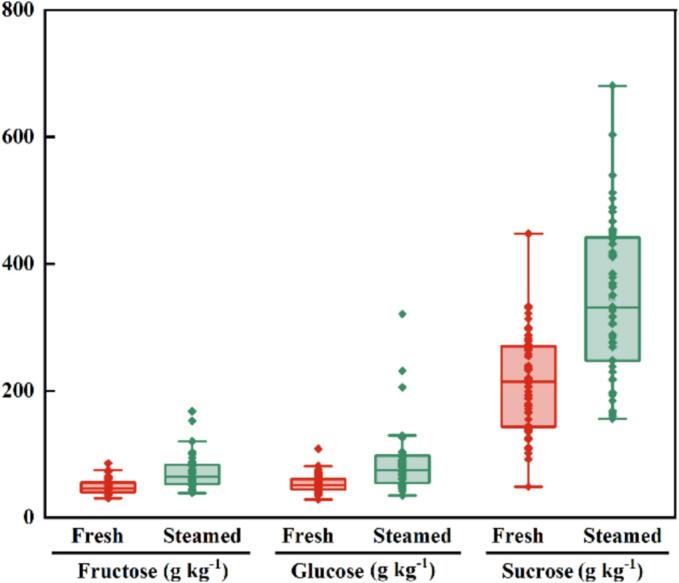
Effects of steaming treatment on Fructose, Glucose, and Sucrose content in jujube fruits of different cultivars.

The changes in sugar content (fructose, glucose, and sucrose) across all cultivars after steaming are summarized in Fig. 5, with detailed data provided in Table S4.

Fructose, a primary monosaccharide in jujube fruit, contributes both to sweetness and to physiological functions such as energy metabolism and osmoregulation. Steaming significantly increased fructose content across all cultivars (Fig. 5). Mean values rose from 48.86 ± 1.19 g kg^−1^ (fresh) to 72.29 ± 1.99 g kg^−1^ (steamed), representing a 47.95 % increase. Among cultivars, JXMZ showed the most pronounced increase (from 38.89 to 152.57 g kg^−1^, a 292.31 % rise), whereas CHZ displayed the least change (from 40.05 to 40.65 g kg^−1^, a 1.50 % increase). This increase may be attributed to the high-temperature, high-humidity conditions of steaming, which promote the release of free fructose. Additionally, partial sucrose hydrolysis into fructose and glucose under thermal conditions further contributed to fructose accumulation (Mei et al., 2025).

Glucose, another key monosaccharide in jujube fruit, not only contributes to energy metabolism but also plays an essential role in fruit ripening and storage. Steaming significantly increased glucose content across all cultivars (Fig. 5). Mean values rose from 50.38 ± 1.93 mg/g (fresh) to 86.13 ± 2.94 g kg^−1^ (steamed), representing a 70.96 % increase. Among cultivars, XT0605 showed the largest increase (from 50.04 to 321.01 g kg^−1^, a 541.51 % rise), whereas LYX exhibited the smallest change (from 66.42 to 69.04 g kg^−1^, a 3.94 % increase). The universal rise in both fructose and glucose can be attributed primarily to sucrose hydrolysis, the main disaccharide in jujube, under the influence of heat and potentially endogenous acids or enzymes (Guo et al., 2015; Xu et al., 2023). In addition, high-temperature-induced cell wall disruption may facilitate the release of bound glucose, further contributing to its accumulation (Zeng, Li, Yang, Lv, & Xiao, 2025).

Sucrose, the primary disaccharide in jujube fruit, functions as both an energy source and a key regulator of flavor, texture, and storage stability. After steaming, the sucrose content exhibited cultivar-dependent variability (Fig. 5). Some cultivars, such as SDZ (from 110.61 to 368.99 g kg^−1^, a 233.60 % increase) and NJDMZ (from 48.77 to 167.95 g kg^−1^, a 244.37 % increase), showed significant increases. These changes may result from the breakdown of complex carbohydrates (e.g., raffinose-family oligosaccharides) into sucrose or differential inactivation of invertase enzymes among cultivars. In contrast, other cultivars, including HBLXZ (from 238.80 to 247.67 g kg^−1^, a 3.71 % increase) and CXDZ (from 193.38 to 197.19 g kg^−1^, a 1.97 % increase), showed little change, possibly due to differences in cellular density or the presence of protective matrix components.

The increase in sugars, particularly reducing sugars such as fructose and glucose, fundamentally alters the sensory properties of steamed jujube. The marked accumulation of simple sugars directly accounts for the enhanced sweetness, a major driver of consumer preference. Cultivars with the greatest post-steaming sugar accumulation (e.g., FMG, X13YZ) are therefore well suited for direct consumption as sweet snack products.

### 3.6 Antioxidant activity

The antioxidant capacities of 52 jujube cultivars are summarized in Table S5. Both ABTS free radical scavenging capacity and hydroxyl radical scavenging capacity showed declines after steaming. For ABTS radical scavenging activity, steaming led to a substantial reduction, with mean values decreasing from 53.94 % (fresh) to 36.02 % (steamed). This decline is consistent with the observed losses of phenolic compounds and vitamin C, highlighting the thermal degradation of heat-sensitive antioxidants (Zeng et al., 2025). In contrast, hydroxyl radical scavenging activity showed greater heat tolerance, decreasing only from 79.10 % to 70.07 % on average after steaming. This relative stability may be explained by contributions from heat-stable antioxidants, such as carotenoids, as well as Maillard reaction products (MRPs) formed during steaming (Bassey, Cheng, & Sun, 2024; Nie, Chen, Lu, & Dai, 2021). Notably, JXMZ and MLC retained exceptionally high hydroxyl radical scavenging activity (>85 % in fresh samples and > 75 % in steamed samples), making them promising candidates for functional food development where antioxidant potency is a key quality attribute.

### 3.7 Affiliation function analysis

Color parameters (L*, a*, b*), textural properties (hardness, springiness, adhesiveness, cohesiveness), and other indicators were evaluated using the affiliation function method, and the average affiliation function value was calculated for each cultivar (Li, Gu, et al., 2023). As shown in Table S6, the steamed cultivar with the highest average value was PZ, while JZ60 had the lowest. The top 10 cultivars, ranked in descending order, were PZ, NJDMZ, JXMZ, XZ, FS, SXBP, QXYZ, WD, X13YZ, and MSZZ. By integrating sensory evaluation scores, a comprehensive assessment of steamed jujube quality identified WD, X13YZ, and JXMZ as outstanding cultivars. These cultivars effectively balance the often-competing demands of processing by maintaining favorable texture, retaining nutrients above average, and achieving high sensory acceptance. Such traits make them strong candidates for commercial-scale steamed jujube production.

### 3.8 Correlation analysis and hierarchical cluster analysis

**Fig. 6:**
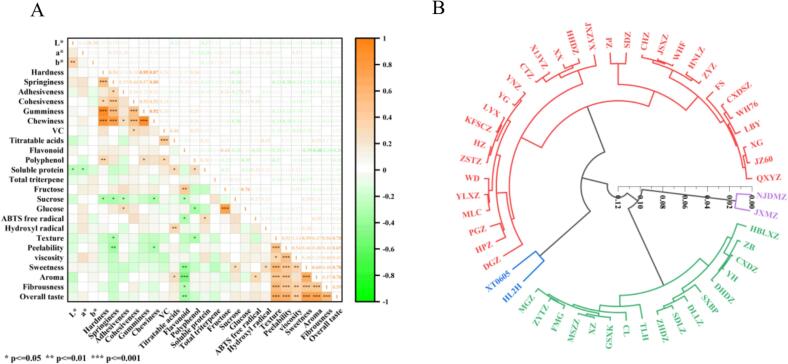
(A) Heatmap of the correlation between quality indicators and sensory evaluation. (B) Clustering dendrogram of jujube fruits of 52 cultivars. Significance levels in (A): *P < 0.05, **P < 0.01, **P < 0.001 (Pearson correlation).

To clarify the contributions of physicochemical and nutritional properties to sensory quality, a correlation heatmap was generated to visualize relationships between key quality indicators and sensory attributes of steamed jujube (Fig. 6A). Hardness showed a very strong positive correlation with gumminess and chewiness, confirming its role as a primary determinant of these texture parameters, consistent with previous findings (Wang, Zhang, et al., 2025; Yang et al., 2025). Overall taste correlated strongly with sweetness, aroma, and texture, underscoring that sensory perception is shaped by multiple interacting attributes (Boasiako, Yinka, Yuqing, Boateng, & Ma, 2024). Fructose and glucose were strongly correlated, reflecting their coordinated accumulation within metabolic pathways. In contrast, titratable acidity exhibited a negative correlation with sweetness perception, highlighting the importance of the sugar–acid ratio in determining flavor balance. Taste scores supported these relationships: high-sugar cultivars such as X13YZ and WD achieved significantly higher overall taste scores than low-sugar cultivars such as PGZ and DGZ, confirming the positive role of sugar content in sensory pleasantness. Importantly, a trade-off was identified, while monosaccharides like fructose enhance sweetness, elevated sucrose levels were associated with excessive softening, indicating that processing optimization requires balancing sweetness with structural integrity. In addition, soluble protein content showed a positive correlation with both ABTS and hydroxyl radical scavenging capacity, suggesting that protein fractions contribute dual functions, serving as both nutritional and antioxidant components.

Based on the physicochemical characteristics and sensory evaluation data of 52 cultivars, a dendrogram was generated using hierarchical cluster analysis (Ward's method; Euclidean distance), revealing four distinct quality groups (Fig. 6B). Each class exhibited unique physicochemical and sensory profiles: Class I included two cultivars (XT0605 and HL2H, 3.85 % of samples). These were characterized by pronounced antioxidant activity, moderate sweetness, and firm texture. Their mechanical stability and bioactive compound content make them well-suited for functional health foods, such as antioxidant-enriched dried jujube, low-sugar preserves, and nutraceutical jams. Class II contained 31 cultivars (59.62 % of samples; e.g., QXYZ, JZ60, XG, FS, PZ, WD, PGZ, HPZ). This heterogeneous group exhibited polarized taste profiles, wide variability in sweetness, and inconsistent texture. To optimize utilization, grading-based processing is recommended: premium cultivars for high-value snacks (e.g., chocolate-coated jujube) and lower-grade cultivars for industrial applications (e.g., flavoring pastes, functional additives), thereby reducing waste. Class III comprised two cultivars (NJDMZ and JXMZ, 3.85 % of samples). These showed excellent mechanical stability and high triterpene acid content. Their strong thermal stability and bioactive profiles make them ideal for medicinal products (e.g., triterpenoid capsules) or heat-resistant foods (e.g., retort-stable canned jujube). Class IV included 17 cultivars (32.69 % of samples; e.g., MGZ, FMG, XZ, ZHDZ, SXBP, ZB). These were characterized by high fructose levels, outstanding sweetness, and rich flavor, making them suitable for fermented products and natural sweeteners (e.g., jujube wine, vinegar, and fruit-based glucose syrup) (Saboktakin-Rizi, Alizadeh Behbahani, Hojjati, & Noshad, 2021). This hierarchical clustering provides a practical framework for industrial application of jujube germplasm, enabling targeted cultivar selection to maximize product value while minimizing resource waste—a critical advantage for modern agricultural processing.

## 4 Conclusion

This study systematically evaluated the effects of steaming on the quality characteristics of 52 jujube cultivars using multidimensional analyses. Steaming markedly altered color (decreased *L* and *a* values, increased *b* values) and texture (reduced hardness, increased adhesiveness). Nutritional profiling revealed substantial losses of heat-sensitive components (vitamin C, flavonoids, phenols), while titratable acidity and total triterpenes increased in certain cultivars. Sugar metabolism analysis showed significant accumulation of fructose and glucose, with cultivar-dependent variation in sucrose dynamics.

Moreover, integrating the affiliation function and hierarchical clustering, WD, X13YZ, and JXMZ were identified as the most suitable cultivars for industrial steaming. A four-class classification framework (Classes I-IV) was also established, offering targeted strategies for cultivar utilization.

Overall, these findings provide a robust theoretical basis and practical decision-making tool for optimizing steaming-based processing of jujube. This approach supports cultivar selection to enhance nutritional value and sensory quality, thereby maximizing the industrial and consumer potential of jujube germplasm resources.

## CRediT authorship contribution statement

**Hanbing Zhu:** Writing – review & editing, Writing – original draft, Formal analysis. **Jia Tian:** Validation, Investigation, Data curation. **Junguang Ning:** Validation, Investigation, Data curation. **Ruirui Dao:** Validation, Investigation, Data curation. **Fuxu Pan:** Visualization, Methodology, Conceptualization. **Mingrui Chen:** Visualization, Methodology, Conceptualization. **Zhuanzhuan Liu:** Visualization, Methodology, Conceptualization. **Mingzhu Lu:** Visualization, Methodology, Conceptualization. **Mengjun Liu:** Project administration. **Changwei Ao:** Validation, Supervision, Data curation. **Zhihui Zhao:** Writing – review & editing, Supervision, Project administration.

## Declaration of competing interest

The authors declare that they have no known competing financial interests or personal relationships that could have appeared to influence the work reported in this paper.

## Data Availability

Data will be made available on request.

## Appendix A Supplementary data

Supplementary materials Image 1
